# Overexpression Cathepsin D Contributes to Perineural Invasion of Salivary Adenoid Cystic Carcinoma

**DOI:** 10.3389/fonc.2018.00492

**Published:** 2018-10-31

**Authors:** Mei Zhang, Jia-shun Wu, Xiao Yang, Xin Pang, Li Li, Sha-sha Wang, Jing-biao Wu, Ya-jie Tang, Xin-hua Liang, Min Zheng, Ya-ling Tang

**Affiliations:** ^1^State Key Laboratory of Oral Disease, West China Hospital of Stomatology, Sichuan University, Chengdu, China; ^2^National Clinical Research Center for Oral Diseases, West China Hospital of Stomatology, Sichuan University, Chengdu, China; ^3^Department of Oral and Maxillofacial Surgery, West China Hospital of Stomatology, Sichuan University, Chengdu, China; ^4^Department of Stomatology, Zhoushan Hospital, Wenzhou Medical University, Zhoushan, China; ^5^Key Laboratory of Fermentation Engineering (Ministry of Education), Hubei Provincial Cooperative Innovation Center of Industrial Fermentation, Hubei Key Laboratory of Industrial Microbiology, Hubei University of Technology, Wuhan, China

**Keywords:** cathepsin D (CTSD), salivary adenoid cystic carcinoma (SACC), perineural invasion (PNI), invasion frontier, cytoskeletal organization

## Abstract

**Objective:** Cathepsin D (CTSD) is a pivotal orchestrator in the occurrence and development of tumors. Recently, CTSD was detected in salivary adenoid cystic carcinoma (SACC). However, its functional role in perineural invasion (PNI) of SACC remained elusive. We conducted the present study to detect the expression of CTSD in SACC, analyze the correlation between CTSD expression and prognosis of SACC patients and elucidate the role of CTSD in occurrence of PNI in SACC to lay the foundation for further studies.

**Methods:** Immunohistochemical analysis was conducted to assess CTSD and Ki67 expression in 158 SACC samples and 20 normal salivary gland samples adjacent to carcinoma. Meanwhile, the correlation between CTSD and PNI of SACC specimens was analyzed using Wilcoxon test. QRT-PCR, immunofluorescence and western blot analysis were used to examine the levels of CTSD mRNA and protein in SACC-LM cell line. SiRNA-mediated CTSD silence was performed. Scratch wound healing assay, transwell invasion assay and DRG co-culture assay of PNI was used to detect the ability of migration, invasion and PNI. FITC-phalloidin was used to detect cytoskeletal organization.

**Results:** Our data demonstrated that the positive expression of CTSD was observed in 74.1% (117/158) of SACC cases, and the expression of CTSD was significantly correlated with the PNI (*p* < 0.05). The ability of migration, invasion, and PNI could be inhibited significantly by siRNA-mediated CTSD silence (*p* < 0.01). Furthermore, siRNA-mediated CTSD silence inhibited cytoskeletal organization and pseudo foot formation in SACC-LM cells.

**Conclusion:** Our results suggested that an association between PNI and expression of CTSD existed. CTSD may promote PNI of SACC accompanied by cytoskeletal organization and pseudo foot formation.

## Introduction

Salivary adenoid cystic carcinoma (SACC) is a kind of malignant epithelial tumor, accounting for about 10% of all salivary gland neoplasm and 21~24% of salivary gland malignancies. Although the 5-year disease-free survival rate reaches 90%, it drops to 10% after 20 years owing to local recurrence and hematogenous spread to distant organs (1). PNI and vascular metastasis are the most important biological characteristics of SACC (2).

Perineural invasion (PNI) of malignant tumor is a form of tumor progression, which means cancer cells encroach along nerves. PNI is considered as a marker of poor prognosis for many malignant neoplasm, including head and neck (1, 3), pancreatic (4), prostate (5), colorectal (6), gastric (7), breast (8) cancer, and so on. It hinders curative resection. Residual tumor cells in or around nerves are closely correlated with increased locoregional recurrence of post-operation and decreased survival. Therefore, it is necessary to elucidate the crosstalk between nerve cells and tumor cells and explore the molecular mechanism of tumor cells promoting the process of PNI in SACC.

One of the important mechanisms for PNI is the proteolysis of the extracellular matrix (ECM). A series of studies demonstrated that enzymes such as MMP-2, MMP-9, and uPA generated by tumor cells participated in the proteolytic degradation of the EMC, disrupted the paratumoral anatomy, advanced tumor cells' further penetration, and led to PNI of SACC, eventually (9–11). Cathepsin D (CTSD), a broadly expressed peptidase, belongs to the family of lysosomal aspartic protease, whose most general and basic function is intracellular catabolism in lysosomal compartments (12, 13). Besides, it also has been implicated as an important factor in many physiological and pathological processes, such as neovascularization of endothelial progenitor cells, hormone and antigen processing, cell growth, and tissue homeostasis, the metabolic degradation of proteins and peptides, inflammation, and atherosclerosis (14–16). Recently, a wide array of studies documented that CTSD facilitates invasion and dissemination of tumors via different mechanisms related to its proteolytic activities (17–19), and CTSD was also detected in SACC (20). However, there is little information about the relationship between CTSD and PNI in SACC cases, and the role of CTSD at nerve invasion frontier of SACC remains unknown.

Therefore, the aim of the present study is to examine the expression of CTSD in normal salivary gland and SACC specimens, analyze the relationship between CTSD expression and patients' prognosis and PNI, and explore the effect of CTSD on the abilities of migration, invasion and PNI of SACC-LM cells to elucidate the role of CTSD in PNI of SACC.

## Materials and methods

### Patients and specimens

One hundred and fifty eight patients with SACC who underwent resection of their tumors without preoperative chemotherapy, hormone therapy, or radiotherapy at the Department of Oral and Maxillofacial Surgery, West China Hospital of Stomatology, Sichuan University, between 2002 and 2007 were recruited for the study after giving their informed consents. All of these cases had undergone surgical resection of adenoid cystic carcinoma of the salivary gland. Demographic and other variables, including size and site of primary tumor, dates of diagnoses, PNI, local regional recurrence, and distant metastasis were retrieved from the database provided by the tumor registry. Besides, 20 cases of normal salivary gland adjacent to carcinoma were included in this study. The protocol of the study was approved by the Institutional Ethics Committee of West China Center, Sichuan University, China.

The presence of PNI was assessed in all SACC specimens by two pathologists, who were not given any information on the patients. The SACC specimens with PNI were further divided into two groups: far away nerve site of SACC with PNI group (the largest extension tumor area without nerve under the same 20 high-power field) and nerve invasion frontier of SACC with PNI group (tumor cells infiltrating or around the nerve under the same 20 high-power field). Histopathological subtypes of cribriform, tubular, or solid patterns of each case were recorded based on the World Health Organization's histological classification of salivary gland tumors (21). Tumor stage clinically was determined according to the International Union against Cancer TNM classification of malignant tumors (22). Diagnostic workup for the detection of distant metastasis included conventional chest radiographs, computerized tomography, percutaneous needle aspiration biopsy, bone scan, and magnetic resonance imaging (23). Representative sections from primary tissue were used for immunohistochemical analysis.

### Immunohistochemistry

Anti-Cathepsin D rabbit monoclonal antibody (ab75852, 1:200) was purchased from Abcam (Cambridge, MA, USA). Anti-Ki67 rabbit polyclonal antibody (bs-23102R, 1:400) was purchased in Beijing botson biotechnology co., ltd.

Immunohistochemical detection was performed on a slide carrying 4-mm–thick tissue, which came from the formalin fixed, paraffin-embedded tumor tissue blocks. All sections were dewaxed in xylene and rehydrated in ascending series of ethanol after these sections were baked in a 37 degree oven overnight. The sections were washed in phosphate-buffered saline (PBS) for 5 min twice. Antigen retrieval was carried by citrate antigen retrieval solution (pH = 6.0) in an autoclave for 5 min. Three percent of hydrogen peroxide was incubated for 15 min to block endogenous peroxidase activity and normal goat serum working fluid incubated for 15 min at 37°C after washing 5 min twice and then the sections were exposed to the primary antibodies at 4°C in the wet box for the night. The sections were washed in PBS for 5 min three times and incubated secondary antibody for 15 min. DAB chromogenic reagent was used to detect the reaction of antigen and antibody and the sections were counterstained in hematoxylin, dehydrated in gradient alcohol, cleared in xylene. Negative control groups were performed by substituting PBS for primary antibody.

Quantification of immunohistochemical results were performed by 2 dependent observers, and we observed 8 microscopic fields at 200× magnification per section, and the ratio of positive cells was counted in 1,000 random cells in every field. Semiquantitative classes of immunohistochemical results were observed and analyzed by two independent pathologists. A mean percentage of positive cells were conditional on four areas at 200× magnification per slide, and the percentage was categorized into one of the following groups: 0, <5% (-); 1, 5–25% (+); 2, 25–50% (++); 3, >50% (+++), which represented negative expression, weakly positive expression, moderately positive expression and strongly positive expression, respectively. ++ and +++ mean high expression, and – and + mean low expression. MIB-1 index, which was based on ki67 staining, was used to evaluate the proliferative activity of cells, and an MIB-1 index of 10% is a useful cut-off level.

### Cell line and cell culture

SACC-LM was provided by State Key Laboratory of Oral Diseases & National Clinical Research Center for Oral Diseases, West China Hospital of Stomatology, Sichuan University. SACC-LM was cultured in RPMI-1640 (HyClone, USA) supplemented with 10% fetal bovine serum (FBS), 100 μg/ml ampicillin and 100 μg/ ml streptomycin at 37°C in a humid atmosphere of 5% CO2/95% air.

### siRNA experiments

SACC-LM cells (1 × 10^5^) were seeded in six-well cell culture plates. When cell confluency reached 30–50%, Opti-MEM® I Medium with GlutaMAX™ (Termo Fisher) was mixed separately with 5 μl 20 nM Stealth RNAi™ siRNA (Termo Fisher) and with 5 μl Lipofectamine 2000, incubating 5 min. Above separated mix were combined and incubated for 20 min at room temperature, then 500 μl final mix was added to six-well cell culture plates, adding 1500 μl culture medium at the same time. Medium with Lipofectamine 2000 was removed and replaced with RPMI-1640 with 10% FBS after 6 h. In addition, blank group (only transfection reagent) was used as blank control group. Sequences of siRNA CTSD are as following: sense, 5′-GUGGACCAGAACAUCUUCUTT-3′ and the antisense sequence was 5′-AGAAGAUGUUCUGGUCCACTT-3′. The sequences of negative control are as following: sense, 5′-UUCUCCGAACGUGUCACGUTT-3′ and the antisense sequence was 5′-ACGUGACACGUUCGGAGAATT-3′. All experiments were performed in triplicate.

### Immunofluorescence

For fluorescent immunocytochemistry, the SACC-LM cells were cultured in 24-well cell culture plates for 24 h at 37°C in a humidified atmosphere containing 5% CO_2_. Then cells were fixed for 20 min with 4% paraformaldehyde and were permeabilized with PBS containing 0.2% Triton X-100 for 15 min. Normal goat serum working fluid incubated for 30 min at 37°C and incubated with primary antibody overnight at 4°C, and then staining was detected with fluorescein-conjugated secondary antibodies (PeproTech; 1:200) for 1 h at room temperature in dark condition. After washing with PBS for 3 times, nuclear was stained in 4′, 6-diamidino-2-phenylindole (DAPI) for 5 min. Immunofluorescence signals were examined using a fluorescence microscope (Leica, Germany). SACC tissues were cut into 8-μm thick sections, kept at −80°C until use. Sections were permeabilized with 0.5% Triton X-100 and pre-blocked with normal goat serum working fluid at 37°C for 30 min. Anti-Cathepsin D rabbit antibody (ab75852, 1:200, Abcam) was incubated, and sections were visualized after fluorescein-conjugated secondary antibodies and DAPI were stained.

### Western blotting

Total proteins were extracted from the cells with a total protein extraction kit (Keygen, Nanjing, China), and protein concentrations were detected by a BCA Protein Assay kit (Beyotime, Shanghai, China). After boiled for 5 min at 95°C, 50 μg of each protein sample was electrophoresed on a 12% SDS-polyacrylamide gel and then transferred to a polyvinylidenefluoride (PVDF) membrane. After blocking with 5% bovine serum albumin (BSA), the membrane was incubated with rabbit monoclonal antibody to cathepsin D (ab75852, 1:1000, Abcam) and GAPDH (ab8245, 1:1000, Abcam) at 4°C overnight. The protein was then incubated with goat anti-rabbit secondary antibody (1: 3,000) at room temperature. The membrane was immersed in electrochemiluminescence (ECL) solution, exposed and photographed in a dark room.

### Quantitative real-time PCR (qPCR)

Total RNA was extracted from cells using Trizol reagent (Takara, Tokyo, Japan) according to the manufacturer's protocol. Concentration and quality of RNA were measured with the NanoDrop ND-1000 Spectrophotometer (Thermo Scientific Inc., Waltham, MA). The extracted RNA was then reverse-transcribed into cDNA using SuperScript™ III First-Strand Synthesis System (Invitrogen, Carlsbad, CA). CTSD expression level was assessed using 2 μL of cDNA by SYBR green qPCR SuperMix (Applied Biosystems Life Technologies, Foster, CA, USA) and the primer sequences were as following: forward, 5′-GCCAGGACCCTGTGTCG-3′ and the antisense primer sequence was 5′-GCACGTTGTTGACGGAGATG-3′. GAPDH served as an internal reference and the primer sequences were as following: forward, 5′-CTTTGGTATCGTGGAAGGACTC-3′ and the antisense primer sequences was 5′-GTAGAGGCAGGGATGATGTTCT-3′. Each experiment was conducted in triplicate.

### Scratch wound healing assay

Cells were cultured in 6-well plates. When the cells reached approximately 100% confluence, a 100 μL pipette tip was used to create a vertical linear scratch in the cell plate. The detached cells were removed by PBS washing. The cells were then added to a serum-free culture medium to continue the culturing process. The cell culture plates were observed and photographed at 0, 24, and 48 h. Cell migration was assessed by measuring gap size through using Image-Pro Plus Analysis software (Media Cybernetics company, Rockville, Maryland). All experiments were conducted for 3 times to obtain the average value.

### Transwell invasion assay

Invasion assays were carried out in 24-well culture plate by inserting the transwell chamber covered with Matrigel (pore size, 8.0 μm; BD Biosciences). 1 × 10^5^ SACC-LM cells suspended in 200 μl of serum-free RPMI-1640 were placed in the upper chamber. 600 μl of RPMI-1640 containing 10% FBS were placed in the lower chamber. Cells remaining on the upper chamber were removed using a cotton swab after being incubated at 37°C for 48 h, while cells traversed to reverse face of the membrane were fixed in 4% paraformaldehyde and stained with 0.1% Crystal Violet. Cell invasion was determined by counting stained cells in five fields per chamber. Each experiment was conducted for 3 times.

### *In vitro* DRG co-culture assay of PNI

To study the effects of the CTSD on the PNI activity of SACC cells, we modified the *in vitro* PNI model, based on a technique previously described by Ayala et al. (24–26). Newborn rat dorsal root ganglia (DRG) from the lumbar areas were dissected and implanted in 20 μl of Matrigel gel in the center of the 6-well culture plate. The plate was placed at 37°C for 10 min to allow Matrigel solidification and DRG was fixed in the center of culture plate. Then, RPMI-1640 was added and 1 × 10^4^ cancer cells were seeded far away from DRG. The day after cancer cells were seeded was considered as 1 day. The co-culture model was observed and images were acquired at 1, 3, and 5 day. To perform quantitative analysis of the results, the minimum distance between the cancer cells and DRG was defined. The ability of PNI of cancer cells were determined by counting number of cells around DRG within minimum distance in five fields per well through using Image-Pro Plus Analysis software (Media Cybernetics company, Rockville, Maryland). All experiments were performed for 3 times to obtain the average value. All animal experiments were approved by the Animal Care Committee of Sichuan University, China.

### FITC-phalloidin staining for detecting cytoskeletal organization

Cancer cells were washed twice with PBS, fixed with 4% paraformaldehyde for 15 min, permeabilized with PBS containing 0.2% Triton X-100 for 15 min and blocked with 5% BSA. Then, after FITC-phalloidin (Sigma) was incubated for 30 min, DAPI was used as nuclear staining. All images were visualized and photographed with a fluorescence microscope under the same conditions of excitation and registration. Multiple cells were categorized in each experimental point.

### Statistical analysis

The expression of CTSD in normal salivary gland and different PNI status was evaluated using Wilcoxon test. The correlation between CTSD expression and clinicopathologic parameters was analyzed in all patients through the Wilcoxon test. Overall survival rates were calculated according to the Kaplan–Meier method and differences between the groups were evaluated using the log-rank test. Multivariate analysis was performed by a Cox proportional harzards model to examine the potential prognostic factors. All of the statistical analysis was done using SPSS software package 21.0 (SPSS, Chicago, IL, USA).The results were considered to be significant statistically when P value was <0.05 and comparison between groups was corrected for multiple testing using Bonferroni.

## Results

### Expression and clinical significance of CTSD in SACC

**Figure 1:**
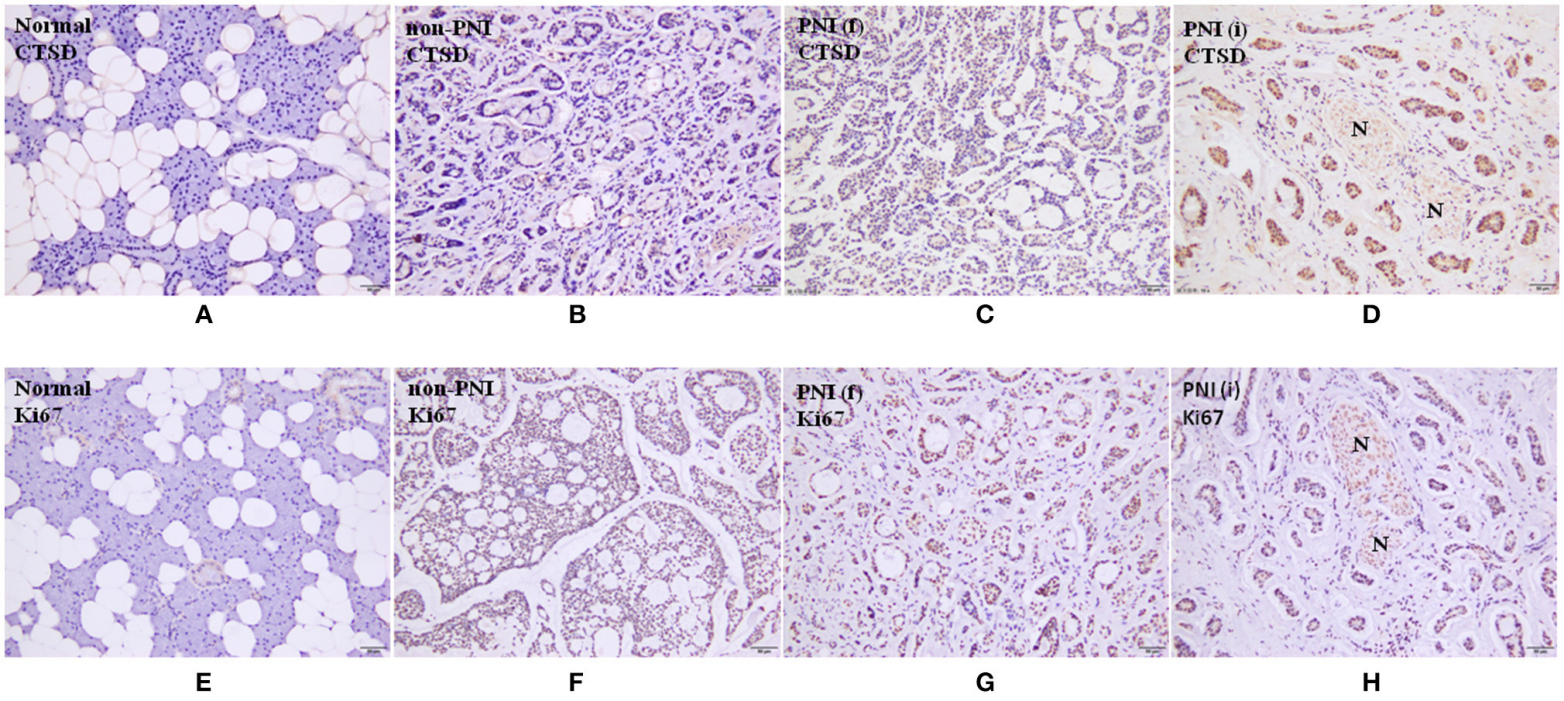
**(A-D)** are immunohistochemical staining of CTSD, (×200). **(E-H)** are immunohistochemical staining of Ki67, (200×). N represents nerve. Normal means normal salivary gland. Non-PNI means non PNI group of SACC. PNI(f) means far away nerve of SACC with PNI group. PNI(i) means nerve invasion frontier of SACC with PNI group.

One hundred fifty eight SACC specimens and 20 normal salivary glands were examined by immunohistochemical staining. CTSD reactivity was generally shown in cytoplasm and only occasionally in the nuclei. The positive expression of CTSD was detected in 74.1% (117/158) of SACC specimens, and the expression of CTSD in 90% (18/20) cases of normal salivary gland was negative and 10% (2/20) cases were weak positive expression. The expression of CTSD in the specimens of SACC was significantly higher than in normal salivary gland (*p* = 0.0001). The positive expression of CTSD in specimens of SACC without PNI (non PNI) group, far away nerve and nerve invasion frontier of SACC with PNI group was 65.4, 81.8, and 87.0%, respectively. The CTSD expression both in far-away nerve and nerve invasion frontier of SACC with PNI group was higher than that in SACC without PNI group, despite the difference of the staining intensity both between non PNI groups and far away nerve of PNI groups, and far away nerve of PNI groups and nerve invasion frontier of PNI groups was not significant (*p* = 0.012, *p* = 0.023) (Table 1; Figure 1). Staining of Ki67 was identified mainly in the nuclei of SACC. The mean MIB-1 index in SACC samples was 30–35%. However, there was no staining of Ki67 in normal salivary gland, which indicated that SACC cells showed a higher proliferative activity than cells of normal salivary gland tissues adjacent carcinoma (Figure 1).

**Table 1:** The expression of Cathepsin D in normal salivary gland and salivary adenoid cystic carcionma.

		**Normal**	**Non-PNI**	**PNI (f)**	**PNI (i)**	***P*-value**
	**Cases**	**(*n* = 20)**	**(*n* = 81)**	**(*n* = 77)**	**(*n* = 77)**	
Cathepsin D	-	18	28	14	10	<0.0001
	+	2	31	33	19	
	++	0	15	14	26	
	+++	0	7	16	22	
	*P* value	0.0001 0.012 0.023				

*Normal, normal salivary gland group*.

*Non-PNI, SACC without PNI group*.

*PNI (f), far away nerve of SACC with PNI group*.

*PNI (i), nerve invasion frontier of SACC with PNI group*.

The correlation between the expression of CTSD and clinicopathologic parameters of SACC was presented in Table 2 There was no significant association of the CTSD positive expression with age, sex, complaint, tumor site, tumor size, involvement of surgical margin, and local regional recurrence (*p* = 0.289, *p* = 0.645, *p* = 0.921, *p* = 0.068, *p* = 0.051, *p* = 0.059, and *p* = 0.064, respectively). CTSD expression was found to be higher in cases of SACC with solid pattern than that with tubular pattern (*p* = 0.0080). However, there was no significant difference existed between cribriform pattern and tubular pattern, cribriform pattern, and solid pattern (*p* = 0.140, *p* = 0.277, respectively). And CTSD expression was significantly correlated with clinic stage and distant metastasis (*p* = 0.005, *p* = 0.001, respectively).

**Table 2:** Correlation between Cathepsin D expression and clinicopathological parameters in salivary adenoid cystic carcinoma.

**Clinicopathological parameters**		**Cases**	**Cathepsin D expression**				***P*-value**
			**-**	**+**	**++**	**+++**	
		158	41	47	41	29	
Ages (years)	<50	73	17	21	19	16	0.289
	≥50	85	24	26	22	13	
Sex	Male	85	26	23	17	19	0.645
	Female	73	15	24	24	10	
Complaint (months)	<12	83	21	27	19	16	0.921
	≥12	75	20	20	22	13	
Site	Minor salivary gland	108	19	38	33	18	0.068
	Major salivary gland	50	22	9	8	11	
Tumor diameter (cm)	≤1	24	9	7	5	3	0.051
	1~2	45	14	14	13	4	
	≥2	89	18	26	23	22	
Clinical stage	I+II	70	24	23	15	8	0.005
	III+IV	88	17	24	26	21	
Histological subtype	Cribriform	72	21	19	13	19	0.044
	Tubular	49	16	16	14	3	
	Solid	37	4	12	14	7	
Involvement of surgical margin	Affect	48	7	12	23	6	0.059
	free	110	34	35	18	23	
Local regional recurrence	Positive	41	8	10	12	11	0.064
	Negative	117	33	37	29	18	
Distant metastasis	Positive	46	7	12	9	18	0.001
	Negative	112	34	35	32	11	

**Figure 2:**
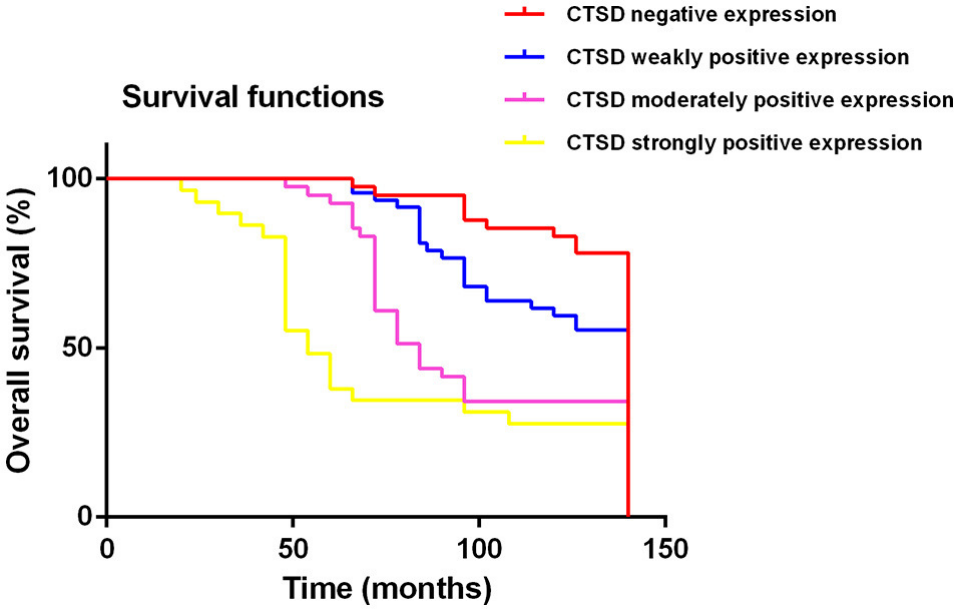
Kaplan-Meier survival analysis on CTSD negative expression, CTSD weakly positive expression, CTSD moderately positive expression, CTSD strongly positive expression group in the patients with SACC (log-rank test, *p* < 0.0001).

The follow-up information was up-dated on December 31, 2017. The median follow up of these patients was 83.7 months (range 3.5–140 months). Kaplan-Meier curves were computed and compared in SACC patients with different CTSD expression status by using the log-rank test (Figure 2) and the result showed that overall survival (OS) time for patients with high CTSD expression was significantly shorter than that without or with low CTSD expression (*p* < 0.0001) The difference between CTSD weakly positive expression group and CTSD moderately positive expression group, CTSD moderately positive expression group, and CTSD strongly positive expression group was significant (*p* = 0.0007 and *p* = 0.0012, respectively), however, the P value between CTSD negative expression group and CTSD weakly positive expression group was not significant (*p* = 0.0169), Multivariate survival analysis for prognostic factors by using a Cox Regression revealed that CTSD expression, clinical stage, involvement of surgical margin, and distant metastasis were independent and significant prognostic factors in this study (*p* < 0.0001, *p* = 0.004, *p* = 0.007, and *p* = 0.006, respectively) (Table 3).

**Table 3:** Multivariate survival analysis for prognostic factors of SACC patients using a Cox regression.

	**B**	**SE**	**Wald**	**df**	**Sig**.	**Exp(B)**	**95.0% CI for Exp (B)**	
							**Lower**	**Upper**
Clinical stage	0.796	0.280	8.100	1	0.004	2.217	1.281	3.835
Involvement of surgical margin	0.639	0.236	7.310	1	0.007	1.894	1.192	3.009
Local regional recurrence	0.384	0.268	2.060	1	0.151	1.469	0.869	2.482
Distant metastasis	0.663	0.243	7.421	1	0.006	1.941	1.204	3.128
Cathepsin D expression	0.560	0.118	22.625	1	0.000	1.750	1.390	2.204

### Expression of CTSD in SACC tissues and SACC-LM cells

**Figure 3:**
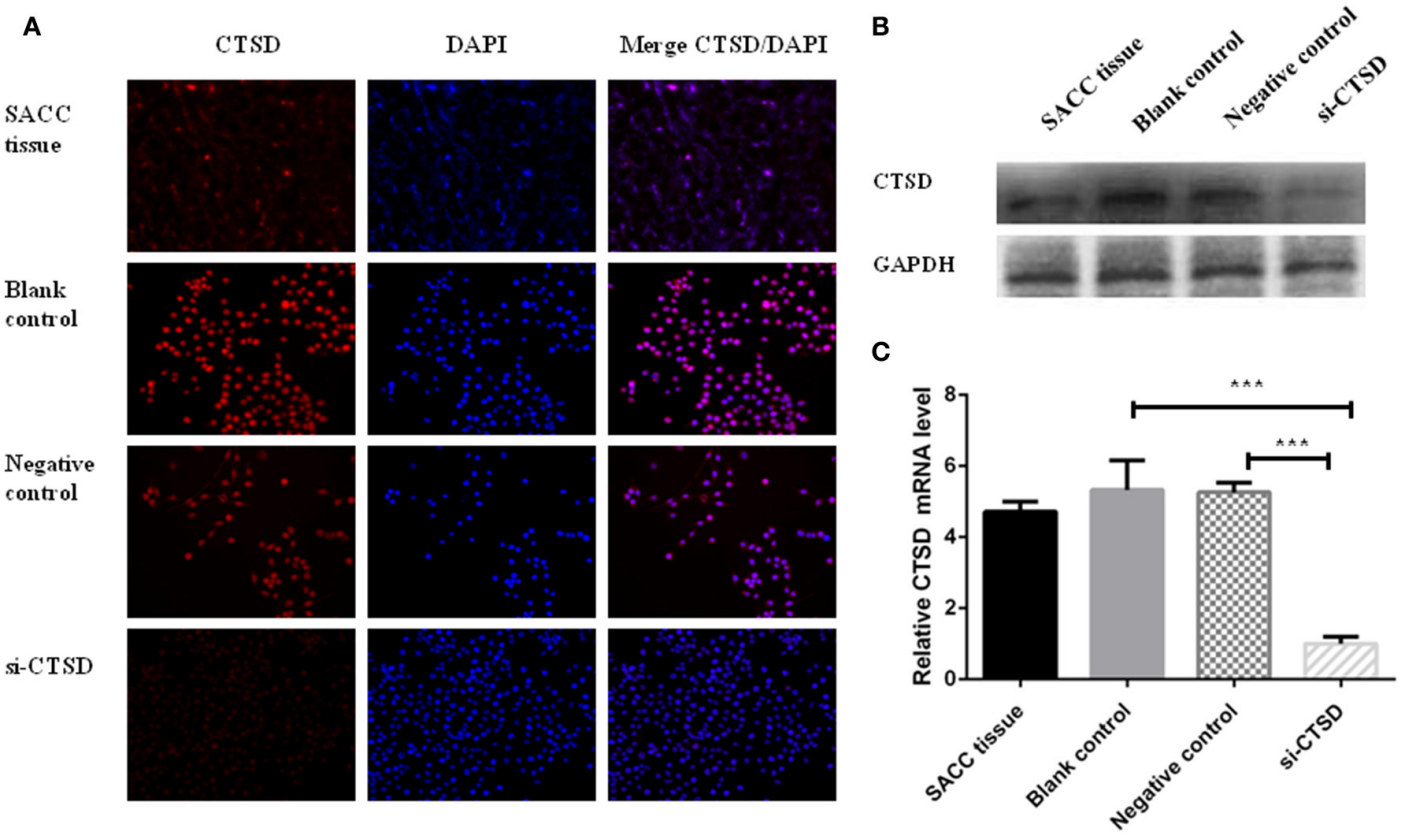
Expression of CTSD in SACC tissues and SACC-LM cells. **(A)** immunofluorescence analysis of CTSD expression in SACC tissue and transfection efficiency of siRNA mediated CTSD knockdown in SACC-LM cells (blank control, negative control, and si-CTSD group), (200×). (**B**) Western blot analyses of CTSD expression in SACC tissue and transfection efficiency of siRNA mediated CTSD knockdown in SACC-LM cells (blank control, negative control, and si-CTSD group). (**C)** qRT-PCR analyses of CTSD expression in SACC tissue and transfection efficiency of siRNA mediated CTSD knockdown in SACC-LM cells (blank control, negative control, and si-CTSD group), (***p < 0.001).

Our results showed that CTSD expression existed by immunofluorescence in SACC tissue and SACC-LM cell line. And the expression of CTSD was dramatically decreased by siRNA treatment compared with blank control group and negative control group in SACC-LM (Figure 3A), and the silence efficiency was also confirmed by western blot and qRT-PCR (blank control group and si-CTSD group, *p* = 0.0009; negative control group and si-CTSD group, *p* < 0.0001) (Figures 3B,C, ^***^p < 0.001).

### Effect of CTSD on migration, invasion, and PNI of SACC-LM cells

**Figure 4:**
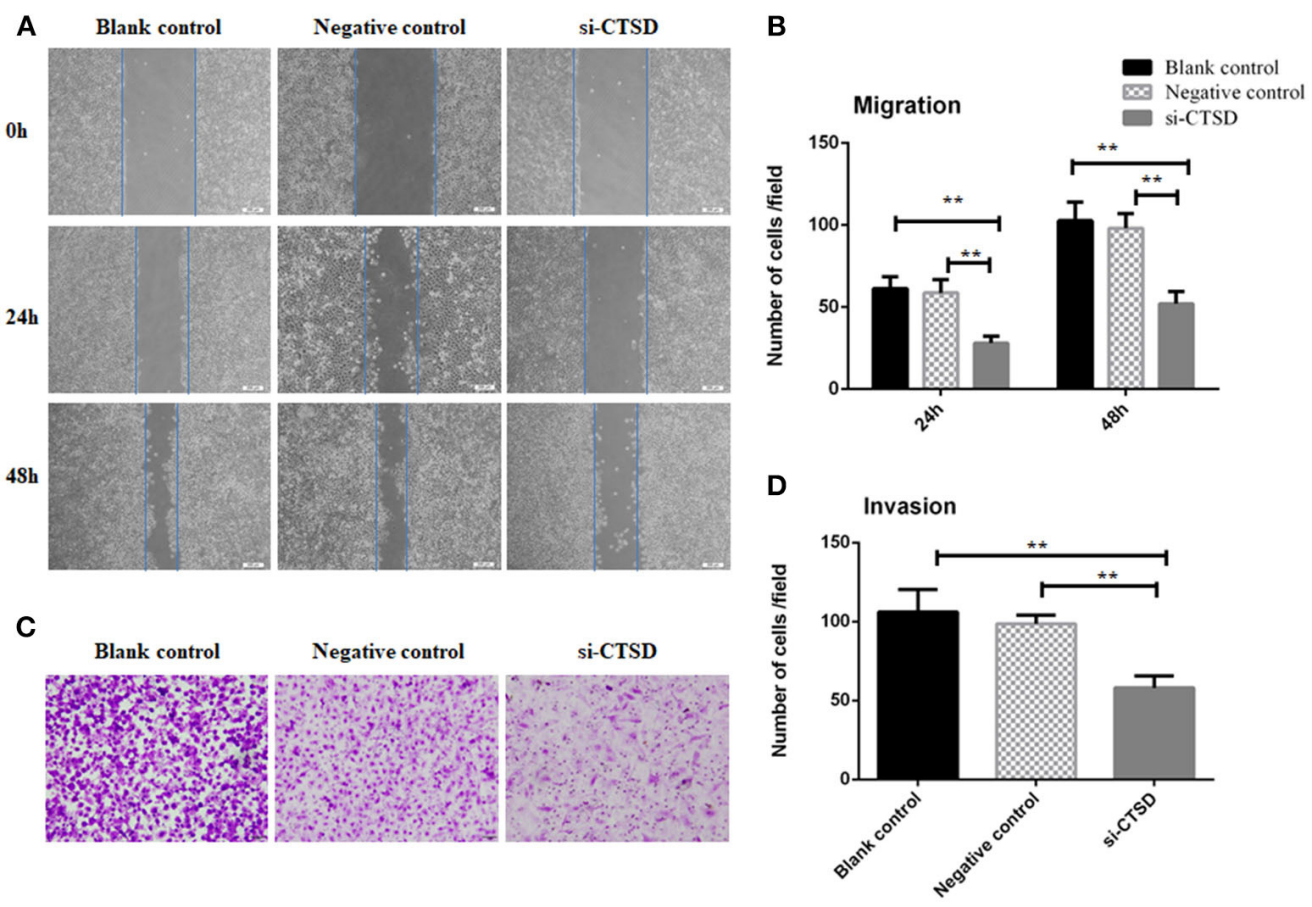
Effect of CTSD on migration and invasion of SACC-LM cells. **(A,B)** Scratch wound healing assay showed that CTSD knockdown inhibited the migratory ability of SACC-LM cells, (200×). **(C,D)** Transwell invasion assay showed that CTSD knockdown inhibited the invasive ability of SACC-LM cells, (200×) (**p < 0.01).

**Figure 5:**
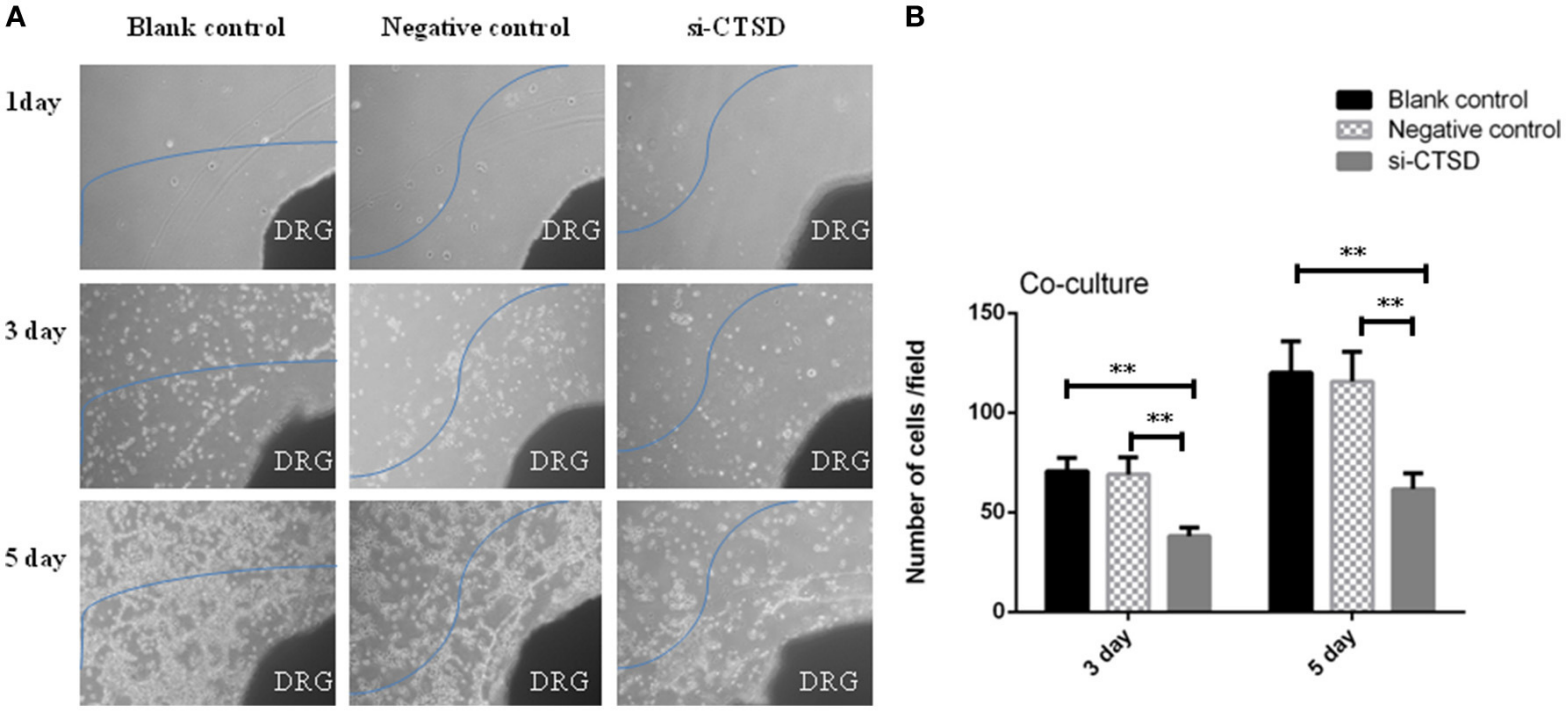
Effect of CTSD on PNI of SACC-LM cells. **(A,B)** *In vitro* DRG co-culture assay of PNI showed that CTSD knockdown inhibited the ability of PNI of SACC-LM cells, (40×) (**p < 0.01). DRG represents newborn rat dorsal root ganglia (DRG).

In scratch wound healing assay, our results showed that CTSD knockdown significantly impaired the migration ability of SACC-LM both at 24 h (blank control group and si-CTSD group, *p* = 0.0023; negative control group and si-CTSD group, *p* = 0.0044) and 48 h (blank control group and si-CTSD group, *p* = 0.0030; negative control group and si-CTSD group, *p* = 0.0024) (Figures 4A,B, ^**^p < 0.01). In transwell invasion assay, silencing CTSD prominently decreased the incidence of invasion in SACC-LM cells (blank control group and si-CTSD group, *p* = 0.0069; negative control group and si-CTSD group, *p* = 0.0017) (Figures 4C,D, ^**^p < 0.01). Furthermore, *in vitro* DRG co-culture assay of PNI, the number of SACC-LM moving toward DRG in siRNA-mediated CTSD silence group significantly decreased compared with blank control group and negative group both at 3 day (blank control group and si-CTSD group, *p* = 0.0021; negative control group and si-CTSD group, *p* = 0.0050) and 5 day (blank control group and si-CTSD group, *p* = 0.0047; negative control group and si-CTSD group, *p* = 0.0054), indicating that CTSD may promote the ability of PNI of SACC-LM cells (Figures 5A,B, ^**^p < 0.01).

### Effect of CTSD on cytoskeletal organization in SACC-LM cells

**Figure 6:**
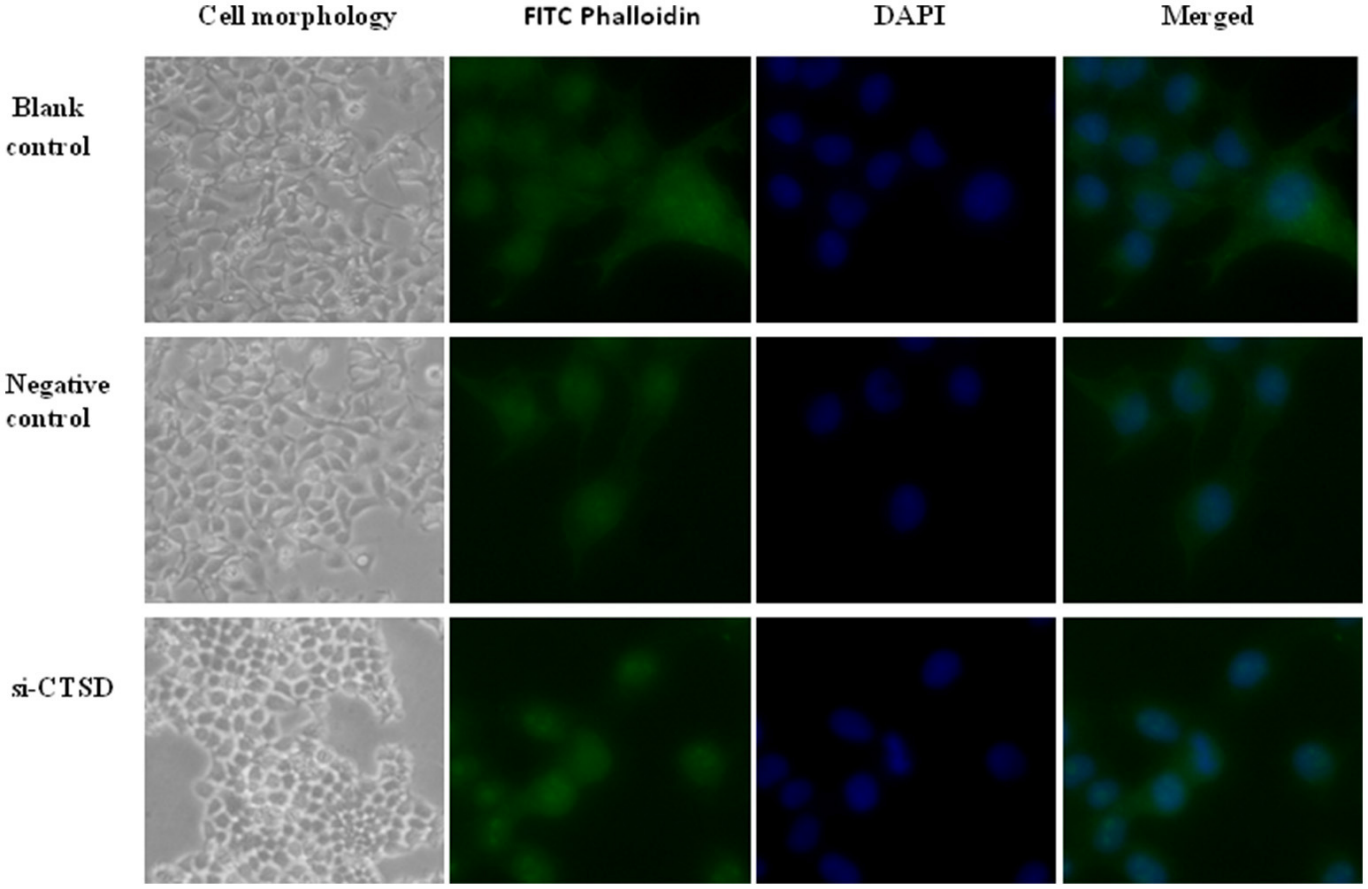
Effect of CTSD on cytoskeletal organization in SACC-LM cells. siRNA-mediated CTSD silence resulted in morphological changes ranging from a migratory, fibroblastoid phenotype toward epithelial plasticity of SACC-LM, (100×). And FITC-phalloidin staining showed that siRNA-mediated CTSD silence led to a collapse in cytoskeletal organization and reduction in pseudo foot formation, (400×).

In our study, SACC-LM cells showed morphological changes through siRNA-mediated CTSD silence, ranging from a migratory, fibroblastoid phenotype toward epithelial plasticity frequently. Besides, we detected the cytoskeleton by using FITC-phalloidin staining and found that compared with blank control and negative control group, siRNA-mediated CTSD silence resulted in a collapse in cytoskeletal organization and reduction in pseudo foot formation. These suggested that CTSD may promote morphological changes, cytoskeletal organization and pseudo foot formation to achieve migration and invasion in SACC-LM cells (Figure 6).

## Discussion

PNI of SACC not only brings about facial paralysis, pain and numbness, but it hinders curative resection. There is a tremendous need to thoroughly explore the nature of PNI of SACC. Here, we examined the expression of CTSD in 158 cases of SACC and 20 cases of normal salivary gland by immunohistochemical analysis and found that higher CTSD expression existed in SACC than that in normal salivary gland. CTSD expression was associated with pattern, clinic stage and distant metastasis. These results were consistent with the previous studies (27). In breast cancer, Achour et al. and Guerra et al. found that CTSD was a marker of poor prognosis and was associated with metastatic relapse of breast cancer (14, 16). Knopfová et al. reported that the elevation of CTSD expression mediated the invasion and metastasis of breast cancer in an organ-specific manner (28), and Anantaraju et al. identified that CTSD inhibitors might be used as potential therapeutics for breast cancer treatment (29). In lung cancer, Giatromanolaki et al demonstrated that CTSD could enhance invasion and was closely related to poor prognosis (30, 31). In ovarian cancer, Pranjol et al. validated that the expression of CTSD existed and it may promote metastasis of tumor cells (32). Gemoll et al. authenticated that increased expression of CTSD facilitated the metastasis of osteosarcomas (17). Chen et al. confirmed that the human olfactomedin 4 gene (OLFM4) could suppress bone metastasis of prostate cancer through inhibiting CTSD (33). Whiteman et al verified that CTSD expression could accelerate the metastatic spread of pancreatic cancer through upregulating S100P (34). Besides, CTSD also played a pivotal modulatory role in invasion process of hepatocellular carcinoma and glioma (35, 36).

In our study, to our knowledge, we first found that CTSD in nerve invasion frontier of PNI group was highly expressed, and the difference between nerve invasion frontier and far away nerve site of SACC specimens was significant. This suggested that CTSD could provide favorable condition for the invasion of SACC cells in the PNI areas, which was in line with the biology characteristic and function of CTSD, which could promote invasion and metastasis through various mechanisms related to its proteolytic activities.

Meanwhile, our *in vitro* assays showed that CTSD could promote SACC-LM cells migration, invasion, and PNI. The reason why CTSD overexpression predicted poor prognosis for SACC patients and promoted PNI was unclear. Recently, considerable researches had showed that cytoskeleton usually connected to the cytomembrane, orchestrated crosstalk between the cell membrane and ECM, and cytoskeleton rearrangement could promote invasion and migration of tumor cell by increasing cell motility (37, 38). In glioma, Gondi et al. validated that siRNA-mediated silence of uPAR and cathepsin B (CTSB) inhibited glioma cell migration and was accompanied by cytoskeletal condensation, and the simultaneous silence of uPAR and CTSB was even more effective at inducing cytoskeletal condensation than uPAR alone (39). Alapati et al. found that knockdown of CTSB using shRNA could inhibit the migration and invasion of glioma cells by curbing the signal for cytoskeletal organization generating migratory arrest (40) and Rao et al. identified that siRNA-mediated CTSB and uPAR down-regulation could inhibit glioma cell adhesion and invasion by down-regulating cytoskeletal proteins talin and vinculin (41). Xiong et al. reported that cathepsin L (CTSL) could promote invasion and migration of human glioma U251 cells by enhancing the activity of cytoskeletal protein, including RhoA and CDC42 (42). In human lung cancer, Han et al. provided evidence that overexpression of CTSL induce A549/DDP and A549/TAX cells to undergo morphological and cytoskeletal changes with increased tumor cells' invasion and migration abilities (43). In prostate cancer, Jevnikar et al. confirmed that Cathepsin H (CTSH) was able to regulate cell migration and invasion via cleaving the cytoskeletal protein talin, which mediated cell migration by activating integrins (44). In our study, we also found that CTSD siRNA treatment led to cell morphological transformation from mesenchymal like cells to epithelioid cells, collapse in cytoskeleton and reduction in pseudo foot formation of SACC-LM. This indicated that CTSD may accelerate migration and invasion through promote cytoskeletal remodeling and pseudo foot formation in SACC-LM cells.

## Conclusion

The present study demonstrated that CTSD contributed to perineural invasion of SACC and was closely associated with the poor prognosis of SACC patients. CTSD may promote migration and invasion via promoting cytoskeletal reorganization and pseudo foot formation of SACC-LM cells. However, the concrete molecular mechanisms of CTSD promoting PNI in SACC remains unclear, and further research is necessary to investigate the biology behaviors and underlying molecular mechanism of tumor cells at nerve invasion front in SACC.

## Ethics statement

The use of human tissue samples and clinical data was approved by the Institutional Ethics Committee of the West China Medical Center, Sichuan University, China (WCHSIRB-D-2012-097, WCHSIRB-D-2017-120). The written informed consents were obtained from participants through their signatures. All procedures involving animal were approved by the Subcommittee on Research and Animal Care (SRAC) of Sichuan University (WCHSIRB-D-2016-149).

## Availability of data and material

The datasets generated during and/or analyzed during the current study are available in the [PUBMED] repository, (Data Sheet 1).

## Author contributions

MeZ edited the manuscript and launched experimental work. JiaW, XY, XP, LL, SW, JinW and Ya-jT launched experimental work. XL reviewed the manuscript. MiZ and Ya-lT collected the patients' information.

### Conflict of interest statement

The authors declare that the research was conducted in the absence of any commercial or financial relationships that could be construed as a potential conflict of interest.
