# Corrigendum: Overexpression Cathepsin D Contributes to Perineural Invasion of Salivary Adenoid Cystic Carcinoma

**DOI:** 10.3389/fonc.2021.693392

**Published:** 2021-06-23

**Authors:** Mei Zhang, Jia-shun Wu, Xiao Yang, Xin Pang, Li Li, Sha-sha Wang, Jing-biao Wu, Ya-jie Tang, Xin-hua Liang, Min Zheng, Ya-ling Tang

**Affiliations:** ^1^ State Key Laboratory of Oral Diseases, West China Hospital of Stomatology, Sichuan University, Chengdu, China; ^2^ National Clinical Research Center for Oral Diseases, West China Hospital of Stomatology, Sichuan University, Chengdu, China; ^3^ Department of Oral and Maxillofacial Surgery, West China Hospital of Stomatology, Sichuan University, Chengdu, China; ^4^ Department of Stomatology, Zhoushan Hospital, Wenzhou Medical University, Zhoushan, China; ^5^ Key Laboratory of Fermentation Engineering (Ministry of Education), Hubei Provincial Cooperative Innovation Center of Industrial Fermentation, Hubei Key Laboratory of Industrial Microbiology, Hubei University of Technology, Wuhan, China

**Keywords:** cathepsin D (CTSD), salivary adenoid cystic carcinoma (SACC), perineural invasion (PNI), invasion frontier, cytoskeletal organization

In the original article, there was a mistake in **Figure 4** as published. In **Figures 4A, C**, we put the wrong picture due to carelessness. The correct **Figure 4** appears below.

**Figure 4:**
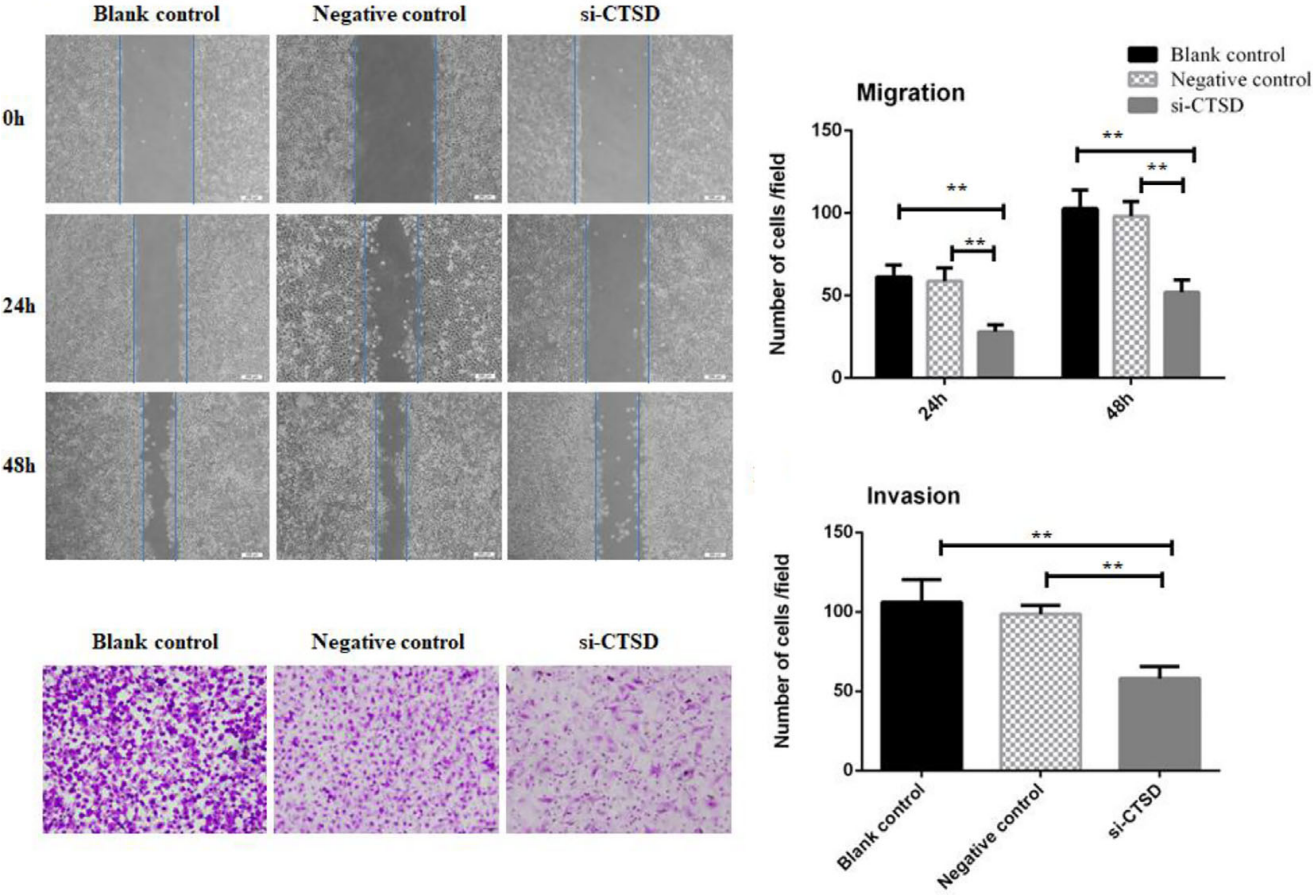
Effect of CTSD on migration and invasion of SACC-LM cells. **(A, B)** Scratch wound healing assay showed that CTSD knockdown inhibited the migratory ability of SACC-LM cells, (200×). **(C, D)** Transwell invasion assay showed that CTSD knockdown inhibited the invasive ability of SACC-LM cells, (200×) (**p < 0.01).

The authors apologize for this error and state that this does not change the scientific conclusions of the article in any way. The original article has been updated.

