# Supplementary material for: Overexpression Cathepsin D Contributes to Perineural Invasion of Salivary Adenoid Cystic Carcinoma
Source: Front Oncol. 2018 Oct 31;8:492. doi: 10.3389/fonc.2018.00492 (PMC6220369; doi:10.3389/fonc.2018.00492)
Supplement: Supplementary file 1 [file Data_Sheet_1.docx]

**Supplementary Table 1. Number of cells in scratch wound healing assay**

|  | Blank control | | | Negative control | | | si-CTSD | | |
| --- | --- | --- | --- | --- | --- | --- | --- | --- | --- |
|  | 1 | 2 | 3 | 1 | 2 | 3 | 1 | 2 | 3 |
| 24h | 69 | 55 | 60 | 66 | 50 | 60 | 25 | 33 | 26 |
| 48h | 106 | 112 | 90 | 88 | 101 | 105 | 51 | 60 | 45 |

**Supplementary Table 2. Number of cells in transwell invasion assay**

|  |  | Blank control | Negative control | si-CTSD |
| --- | --- | --- | --- | --- |
| 24h | 1 | 118 | 105 | 59 |
|  | 2 | 110 | 95 | 65 |
|  | 3 | 90 | 96 | 50 |

**Supplementary Table 3. Number of cells in in vitro DRG co-culture assay of PNI**

|  | Blank control | | | Negative control | | | si-CTSD | | |
| --- | --- | --- | --- | --- | --- | --- | --- | --- | --- |
|  | 1 | 2 | 3 | 1 | 2 | 3 | 1 | 2 | 3 |
| 3day | 78 | 65 | 69 | 77 | 60 | 70 | 35 | 43 | 36 |
| 5day | 126 | 132 | 102 | 108 | 106 | 133 | 61 | 70 | 54 |
